# Supplementary material for: Weakening of the South Asian summer monsoon linked to interhemispheric ice-sheet growth since 12 Ma
Source: Nat Commun. 2023 Feb 14;14:829. doi: 10.1038/s41467-023-36537-6 (PMC9929083; doi:10.1038/s41467-023-36537-6)
Supplement: Supplementary file 1 — Supplementary Information [file 41467_2023_36537_MOESM1_ESM.pdf]

Supplementary material for

Weakening of the South Asian summer monsoon linked to interhemispheric ice-sheet growth since 12 Ma

Zhengquan Yao[a,b,*], Xuefa Shi[a,b,*], Zhengtang Guo[c,d,e], Xinzhou Li[f,g], B. Nagender Nath[h], Christian Betzler[i], Hui Zhang[a,b], Sebastian Lindhorst[i], Pavan Miriyala[j]

[a] Key Laboratory of Marine Geology and Metallogeny, First Institute of Oceanography, Ministry of Natural Resources, Qingdao 266061, China

[b] Laboratory for Marine Geology, Qingdao National Laboratory for Marine Science and Technology, Qingdao 266061, China

[c] Key Laboratory of Cenozoic Geology and Environment, Institute of Geology and Geophysics, Chinese Academy of Sciences, Beijing 100029, China

[d] CAS Center for Excellence in Life and Paleoenvironment, Beijing 100044, China

[e] University of Chinese Academy of Sciences, Beijing 100049, China

[f] State Key Laboratory of Loess and Quaternary Geology, Institute of Earth Environment, Chinese Academy of Sciences, Xi'an 710061, China

[g] CAS Center for Excellence in Quaternary Science and Global Change, Xi'an 710061, China

[h] Geological Oceanography Division, CSIR-National Institute of Oceanography, Dona Paula, Goa 403004, India

[i] Institute of Geology, CEN, University of Hamburg, Hamburg 20146, Germany

[j] CSIR-National Geophysical Research Institute, Hyderabad 500007, India

*Corresponding authors: Z. Yao (yaozq@fio.org.cn), X. Shi (xfshi@fio.org.cn)

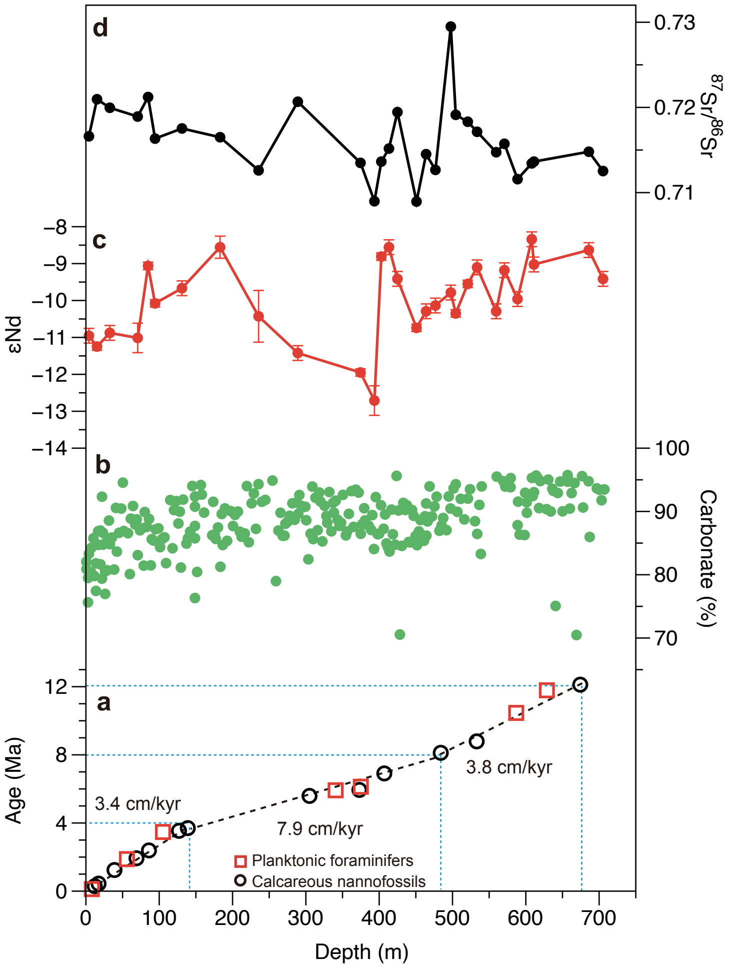

Supplementary Fig. 1. Composite file of Site U1467. a Age-depth profile[7]. **b** Variations in carbonate content[82]. **c, d** εNd (2sigma S.D.) and $^{87}Sr/^{86}Sr$ from the detrital components at Site U1467.

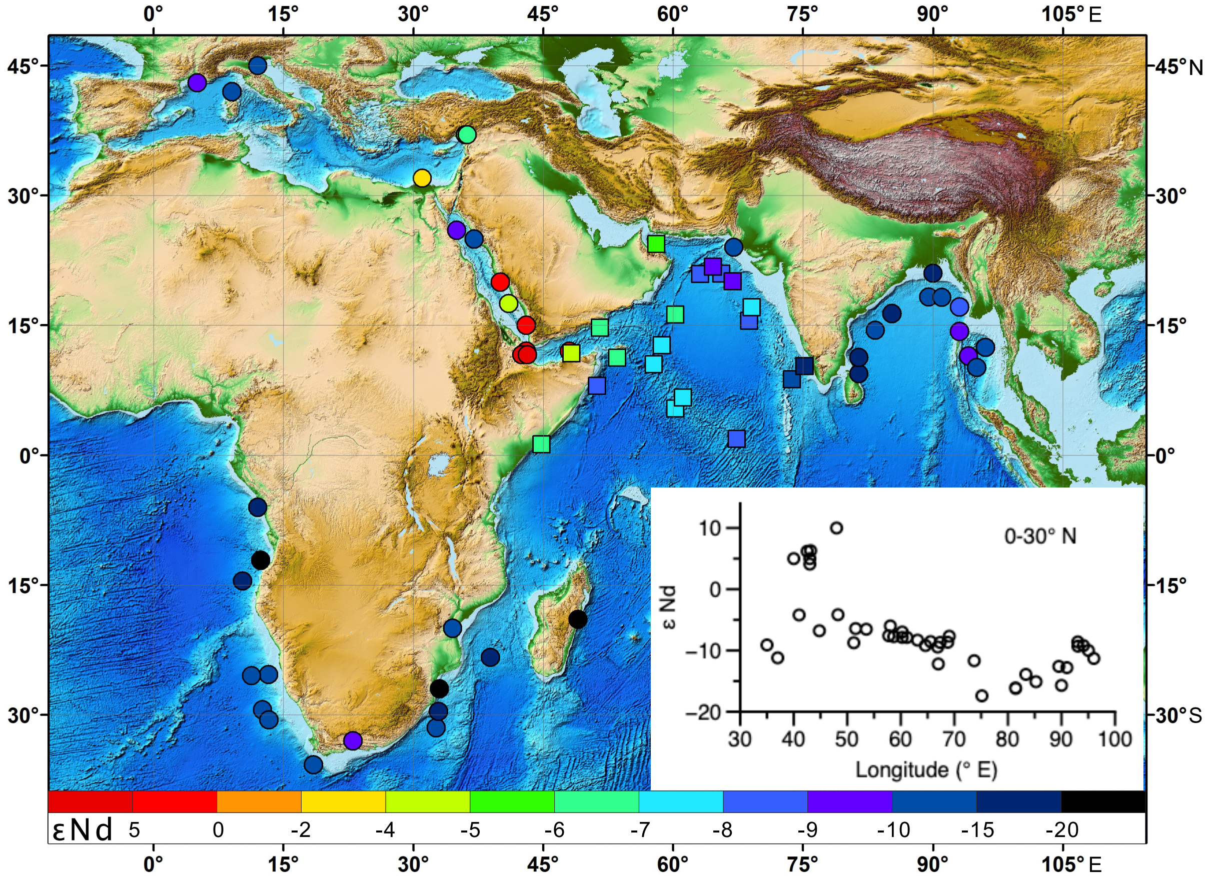

Supplementary Fig. 2. Changes in the εNd values of the core-tope sediments from the Indian Ocean margin. Spatial distribution of εNd values of the core-tope sediments from the Arabian Sea[29] (Squares) and of the marine sediments/rocks along the Indian Ocean continental margin[33] (dots). The εNd values within the regions of 0-30° N are also shown in the bottom panel. The base map is generated using open access software SimpleDEMViewer (http://www.jizoh.jp) with data from https://www.ngdc.noaa.gov/mgg/global/global.html.

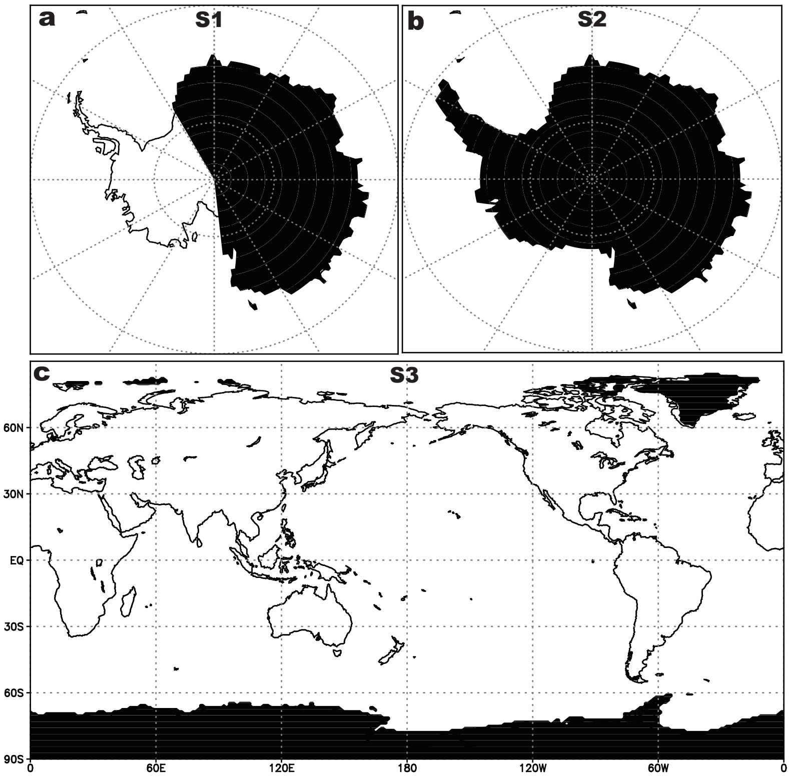

Supplementary Fig. 3. Sketch map illustrating the ice-cover state for three different scenarios. a The East Antarctica is covered by ice (S1). **b** The whole Antarctica is coverd by ice (S2). **c** The ice is present in Greenland superimposed on a constant Antarctic ice-sheet cover (S3). The maps are generated using GrADS[89].

Supplementary Table 1. Isotopic data of $^{86}Sr/^{87}Sr$, $^{143}Nd/^{144}Nd$ and calculated εNd obtained from the detrital components of Site U1467.

Sample ID	Depth (m)	Age (Ma)	$^{87}Sr/^{86}Sr$	$^{143}Nd/^{144}Nd$	εNd
U1467B-2H-1	4.12	0.06	0.71661	0.512077	-10.94
U1467B-3H-2	15.12	0.38	0.72096	0.512062	-11.24
U1467B-5H-1	32.62	1.01	0.71996	0.512081	-10.87
U1467B-9H-1	70.62	1.97	0.71893	0.512074	-11.00
U1467C-10H-2	84.82	2.36	0.72122	0.512174	-9.05
U1467C-11H-2	94.32	2.86	0.71634	0.512122	-10.07
U1467C-16H-2	131.02	3.59	0.71753	0.512143	-9.66
U1467B-21H-5	183.02	4.20	0.71650	0.512200	-8.54
U1467B-27H-2	235.52	4.80	0.71261	0.512104	-10.42
U1467B-32H-6	289.02	5.41	0.72067	0.512053	-11.41
U1467B-44H-3	374.52	6.09	0.71349	0.512026	-11.94
U1467B-46F-3	393.52	6.58	0.70899	0.511987	-12.70
U1467B-48F-3	402.92	6.81	0.71364	0.512187	-8.80
U1467B-50F-4	413.33	7.01	0.71517	0.512200	-8.54
U1467B-53F-2	424.92	7.19	0.71945	0.512156	-9.40
U1467B-58F-4	450.93	7.60	0.70895	0.512088	-10.73
U1467B-61F-3	464.02	7.81	0.71451	0.512111	-10.28
U1467B-63X-4	476.72	8.01	0.71267	0.512119	-10.12
U1467B-65X-5	497.72	8.31	0.72949	0.512137	-9.77
U1467B-66X-3	504.43	8.40	0.71913	0.512108	-10.34
U1467B-68X-1	520.82	8.62	0.71832	0.512149	-9.54
U1467B-69X-3	533.52	8.81	0.71714	0.512172	-9.09
U1467B-72X-1	559.62	9.61	0.71474	0.512111	-10.28
U1467B-73X-2	570.82	9.96	0.71573	0.512168	-9.17
U1467B-75X-1	588.72	10.52	0.71158	0.512128	-9.95
U1467B-77X-1	608.12	11.13	0.71343	0.512211	-8.33
U1467B-77X-3	611.12	11.23	0.71364	0.512176	-9.01
U1467C-35X-1	685.92	12.20	0.71480	0.512196	-8.62
U1467C-37X-1	705.32	12.35	0.71253	0.512156	-9.40
